# CHADS2 scores as a predictor of ischemic stroke after radical prostatectomy

**DOI:** 10.1002/cam4.557

**Published:** 2015-11-21

**Authors:** Yu‐Wan Yang, Shang‐Sen Lee, Chi‐Cheng Chen, Hsin‐Ho Liu, Tsung‐Hsun Tsai, Tien‐Huang Lin, Teng‐Fu Hsieh

**Affiliations:** ^1^ Department of NeurologyChina Medical University HospitalTaichungTaiwan; ^2^ School of MedicineChina Medical UniversityTaichungTaiwan; ^3^ Department of UrologyTaichung Tzu Chi HospitalBuddhist Tzu Chi Medical FoundationTaichungTaiwan; ^4^ School of MedicineTzu Chi UniversityHualianTaiwan

**Keywords:** CHADS2 scores, ischemic stroke, predictor, prostate cancer, radical prostatectomy, risk

## Abstract

Patients with prostate cancer have an increased risk of stroke, but their absolute rate of stroke depends on age and comorbid conditions. The Charlson Comorbidity Index Score (CCIS) is a widely accepted measure for risk adjustment in administrative claims data sets. This study assesses the predictive value of CHADS2 scores and CCIS for stroke among patients with prostate cancer. The study was conducted based on data taken from Taiwan's National Health Insurance Research Database (NHIRD). We identified a total of 5414 participants with nonatrial fibrillation (AF) prostate cancer diagnoses who underwent radical prostatectomy between 1997 and 2011. CHADS2 scores and CCIS were used to stratify the 5‐year ischemic stroke risk. All participants were followed from the date of enrollment until ischemic stroke, death, or the end of the 5‐year follow‐up period. The 5‐year risk of ischemic stroke in the present study was 1.7%. Ischemic stroke has a better correlation with CHADS2 (CHADS2 score = 0 to 1: 0.02%, CHADS2 score = 2 to 3: 13.9%, CHADS2 score ≥ 4: 44.4%; AUC = 0.978) than CCIS (CCIS = 0 to 1: 1.6%, CCIS = 2 to 3: 1.7%, CCIS ≥ 4: 3.8%; AUC = 0.520). Our results show that patients with prostate cancer who underwent radical prostatectomy show significantly higher risk of ischemic stroke in high CHADS2 score patients, and the CHADS2 score could be applied for ischemic stroke prediction. Cardiovascular risks evaluation and management are suggested for prostate cancer patients with higher CHADS2 score.

## Introduction

Prostate cancer is a common noncutaneous malignancy in men. Radical prostatectomy reduces cancer‐specific mortality among men with localized prostate cancer; however, important questions regarding long‐term benefit remain 1, 2, 3. Because prostate cancer is typically diagnosed in elderly men, a significant proportion of patients have comorbidities that result in a high rate of noncancer‐related mortality 3. This raises the need for a test which can identify patients that enjoy long‐term benefits from radical prostatectomy.

An association between cancer and thrombosis has been reported 4, 5, 6, 7, 8. Moreover, substantial experimental data suggest that the coagulation system is implicated in multiple cancer pathways, including tumor proliferation, angiogenesis, apoptosis, and metastasis 9, 10. These reports implied that malignant disease and hemostasis interact to produce thrombosis, leading to ischemic stroke which can result in disability or even death. It is important to determine whether patients who underwent radical prostatectomy for prostate cancer have an elevated risk of ischemic stroke.

The CHADS2 score, which closely correlate major adverse cardiovascular events, is a popular clinical parameter used to assess the risk of ischemic stroke in patients with atrial fibrillation (AF) 11. Various authors have demonstrated that CHADS2 scores are also a good predictor of ischemic stroke in non‐AF patients 12, 13. However, few studies have examined the association between the CHADS2 scores and ischemic stroke in prostate cancer patients. Thus, the correlation of CHADS2 scores and ischemic stroke in non‐AF prostate cancer patients should be evaluated.

Prior studies have shown Charlson Comorbidity Index Score (CCIS) to be useful in predicting mortality for radical prostatectomy patients 14, 15, 16, 17. However, no studies have compared the predictive value for the CHADS2 scores and CCIS for stroke in prostate cancer patients.

We hypothesize that ischemic stroke can be detected by using CHADS2 scores or CCIS in prostate cancer patients who have undergone radical prostatectomy, and test the hypothesis based on prostate cancer patients in Taiwan's National Health Insurance Research Database (NHIRD).

## Materials and Methods

### Ethics statements

The Institutional Review Board of Taichung Tzu Chi General Hospital in Taiwan approved the study protocol (REC103‐43). Because the identification numbers and personal information of the individuals in this study were not included in the secondary files, the review board waived the need for written consent.

### Patients and study design

This study used NHIRD data from 1997 to 2011. Taiwan implemented a National Health Insurance (NHI) program in 1995 to provide comprehensive health care coverage. Enrollment in this government‐run, universal, single‐payer insurance system is mandatory and up to 99% of Taiwan's 23 million residents receive medical care through the NHI program. In addition, >97% of the hospitals and clinics in Taiwan are contracted to provide health care services 18, which are reimbursed by the NIH Bureau. All data related to these services are collected and input into the NHIRD by the National Health Research Institutes to provide a comprehensive record of medical care. The data consist of ambulatory care records, in‐patient care records, and registration files of the insured, and the database includes all claims data from the NHI program 19.

To select potential patients in this cohort, cases of prostate cancer with radical prostatectomy were identified. AF patients were excluded. All patients with newly diagnosed prostate cancer with radical prostatectomy and follow‐up between January 1, 1997 and December 31, 2011 were included. Prostate cancer was defined according to the International Classification of Diseases, Ninth Revision, Clinical Modification (ICD‐9‐CM) code 185 and radical prostatectomy was defined according to NHI Surgical Orders codes 79403B and 79410B. We used the date of radical prostatectomy as the patient's index date. We identified comorbilities and characters of patients from inpatient or outpatient files, at least in three or more consistent diagnoses in outpatient care or once in inpatient care, between the 6 months interval before index date. The scores of patient's comorbilities, such as CHADS2 scores and CCIS, were calculated by these comorbilities. In keeping with previous studies, we did not score prostate cancer as malignancy.

### Research outcomes

The main outcome of the study was the occurrence of ischemic stroke (ICD‐9‐CM Code: 433, 434 and 437.1), which was determined by linking records with inpatient care data of main diagnosis in the NHIRD. If a patient had multiple hospitalizations for ischemic stroke during the study period after index day, only the first hospitalization was studied. This method was used to identify people with ischemic stroke in other studies 12.

### CHADS2 scores

CHADS2 scores were calculated for each patient by assigning one point each for the presence of chronic heart failure, hypertension, age 75 and above, and diabetes. Two points were assigned for history of stroke or transient ischemic attack (TIA) 11. The study patients were divided into four groups by their CHADS2 scores: 0, 1, 2, and ≥3.

### Charlson comorbidity index score

The Charlson Comorbidity Index Score (CCIS) is a widely accepted measure for risk adjustment in administrative claims datasets 20, 21, 22, 23. The CCIS were calculated for each patient by assigning one point each for myocardial infarct, congestive heart failure, peripheral vascular disease, dementia, cerebrovascular disease, chronic lung disease, connective tissue disease, ulcer, chronic liver disease, and diabetes by assigning two points each for hemiplegia, moderate or severe kidney disease, diabetes with end organ damage, tumor, leukemia and lymphoma, three points for moderate or severe liver disease and six points each for malignant tumor, metastasis and acquired immune deficiency syndrome.

### CHA2DS2‐VASc scores

The CHA2DS2‐VASc scores were calculated for each patient by assigning one point each for the presence of vascular disease (history of myocardial infarction, peripheral artery disease, or vascular plaques), age 65–74 years, and sex category (female) and by assigning two points each for congestive heart failure, hypertension, age >75 years, diabetes mellitus, and history of stroke/transient ischemic attack 11.

### Statistical analysis

SPSS version 15 software (SPSS Inc., Chicago, IL) was used for all data analyses. Pearson's chi‐square test was used to assess categorical variables. Continuous variables were analyzed using one‐way analysis of variance (ANOVA).

The receiver operating characteristics curve was used to assess the prediction accuracy for ischemic stroke using CHADS2 scores and CCIS; plots of observed and predicted ischemic stroke were then presented. The cumulative risk of ischemic stroke was estimated using Kaplan–Meier survival curves.

A Cox proportional hazards regression model adjusted for patient characteristics (patients' age, hyperlipidemia, chronic kidney disease, coronary artery disease, socioeconomic status, urbanization, and geographic region.) was used to analyze subsequent incidence of ischemic stroke during the follow‐up period. Hazard ratios (HRs) along with 95% confidence intervals (CIs) were calculated using a significance level of 0.05. Statistical significance was set at a two‐sided *P* < 0.05.

## Results

The NHIRD records 5414 patients diagnosed with prostate cancer undergoing radical prostatectomy from 1997 to 2011 within the NHIRD. Table 1 shows the number, age, gender, and distribution of number of patients in different CHADS2 score and CCIS groups. The mean age at diagnosis was 65 ± 6 years. The mean CHADS2 score was 0.5 ± 0.8 and CCIS was 1.1 ± 1.3. Controls (nonprostate cancer group), two patients for each prostate cancer patient with radical prostatectomy, were randomly selected from the same dataset. These controls did not have a prostate cancer diagnosis and were matched by age by propensity score matching. The average CHADS2 score and CCIS among age matched controls were 0.42 ± 0.94 and 0.01 ± 0.13, respectively (in Fig. S1).

**Table 1:** Baseline characteristics of the patients with prostate cancer and radical prostatectomy from 1997 to 2011 in Taiwan

Variables	*N* (%)
Total	5414
Mean age, years (±SD)	65 ± 6
CHADS2 score	
Mean ± SD	0.5 ± 0.8
0–1	4890 (90.3)
2–3	469 (8.7)
Over 4	54 (1.0)
Charlson comorbidity index score	
Mean ± SD	1.1 ± 1.3
0–1	2851 (52.7)
2–3	2484 (45.9)
Over 4	78 (1.4)
Comborbidities	
Hyperlipidemia	139 (2.6)
Chronic kidney disease	28 (0.5)
Coronary artery disease	240 (4.4)
Socioeconomic status	
Disadvantaged SES	1395 (25.8)
Advantaged SES	4019 (74.2)
Urbanization	
Urban	1995 (36.9)
Nonurban	3418 (63.1)
Geographic region	
Northern and Central	4116 (76.0)
Southern and Eastern	1297 (24.0)

**Figure 1:**
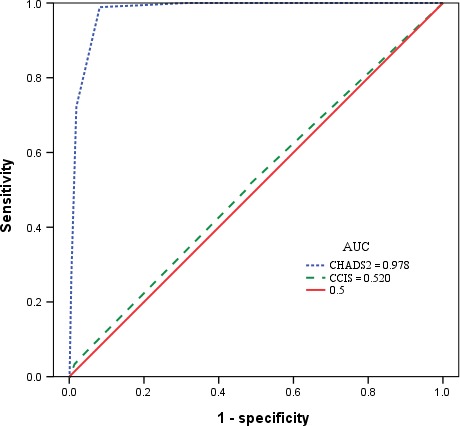
Receiver operating characteristics curve for CHADS2 and Charlson Comorbidity Index Score for the prediction of stroke in prostate cancer patients with radical prostatectomy.

**Figure 2:**
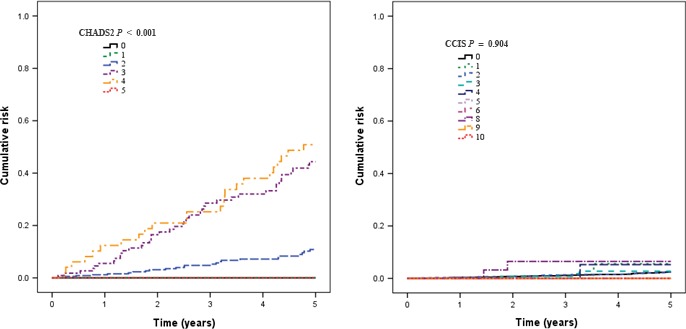
Stroke risk stratified by CHADS2 score and CCIS categories.

We further divided the prostate cancer patients into three groups based on the CHADS2 scores and CCIS (Table 2). As shown in Figure 1 shows, the c‐statistics was 0.978 for the CHADS2 score and 0.520 for CCIS. Figure 2 shows the Kaplan–Meier survival curves for prostate cancer patients with different CHADS2 scores or CCIS. Higher CHADS2 scores or CCIS corresponds with increased chance of ischemic stroke (*P* < 0.001).

**Table 2:** The cumulative rate of stroke in difference CHADS2 and Charlson Comorbidity Index score (CCIS) for 5 years

Variables	*n*	Case (%)	*P*‐value
CHADS2 score			
0–1	4890	1 (0.02)	<0.001
2–3	469	65 (13.9)	
Over 4	54	24 (44.4)	
CCIS score			
0–1	2851	45 (1.6)	0.300
2–3	2484	42 (1.7)	
Over 4	78	3 (3.8)	

In multivariate analysis (Table 3), each additional CHADS2 score is associated with a 4.11‐fold (95% CI, 3.55–4.79) increased risk for ischemic stroke when the CHADS2 score is a continuous variable. In addition, each additional CCIS score is associated with a 1.04‐fold (95% CI, 0.89–1.20) increased risk for ischemic stroke when CCIS score is a continuous variable.

**Table 3:** Hazard ratios of individual CHADS2 and CCIS score for stroke in prostate cancer with radical prostatectomy patients

	Univariate analysis			Multivariate analysis^a^		
	Adjusted HR	95% CI	*P* value	Adjusted HR	95% CI	*P*‐value
CHADS2	3.88	3.40–4.42	<0.001	4.11	3.53–4.79	<0.001
CCIS	1.07	0.92–1.24	0.373	1.04	0.89–1.20	0.635

a Adjust for the patients' age, hyperlipidemia, chronic kidney disease, coronary artery disease, socioeconomic status, urbanization, and geographic region.

## Discussion

The application of CHADS2 scores was found to provide a high prediction accuracy for ischemic stroke in prostate cancer patients who had undergone radical prostatectomy. To the best of our knowledge, this is the first study to demonstrate the usefulness of CHADS2 scores in predicting ischemic stroke in prostate cancer patients.

Our study adds to the existing literature on the utility of CHADS2 scores in predicting ischemic stroke in non‐AF patients with clinical diagnosis of early stage prostate cancer. We provide the general calculation for the rate of incidence as well as a risk assessment for ischemic stroke in cases following radical prostatectomy for prostate cancer. Based on this, clinicians should use CHADS2 scores to identify patients at high risk of ischemic stroke after radical prostatectomy for prostate cancer for aggressive prevention through life style change, diabetes control, and blood pressure management.

Taiwan's National Health Insurance Research Database (NHIRD) has been widely used in epidemiological studies of cardiovascular disease 24, 25. The high accuracy of the NHIRD in recording diagnoses and prescriptions has been reported 24, 25. Several studies have used the NHIRD to investigate associations between different diseases 26, 27, 28. This study benefits from its nationwide, population‐based, cross‐sectional design, with nearly complete follow‐up health care information for the whole study population (99%), as well as the fact that the dataset is routinely monitored for diagnostic accuracy. Furthermore, NHIRD data has been shown to be of high quality (94% accuracy) in cases with a principal diagnosis of ischemic stroke 25, 29.

One population‐base study indicated that, after the introduction of prostate‐specific antigen testing in 1996, men up to age 80 with normal metabolic levels had a 13% risk of prostate cancer, a 2% risk of prostate cancer death, and a 30% risk of death from other causes, whereas men with metabolic aberrations had corresponding risks of 11, 2, and 44% 3. This study indicates the importance of nonprostate cancer death in prostate cancer patients, showing a 1.7% overall risk of ischemic stroke in prostate cancer patients treated with radical prostatectomy. Over the 5‐year follow‐up period, patients in the high‐risk population (CHADS2 ≥ 4) was 44.4%, as opposed to 13.9% for CHADS2 = 2 to 3, and 0.02% for CHADS2 = 1 or 0. Current guidelines for AF suggest that CHADS2 scores are useful in the selection of antithrombotic therapy; therefore, high‐risk patients (CHADS2 scores ≥ 2) may benefit from anticoagulation therapy 11. Based on results from this and other studies, antithrombotic therapy may be essential for high‐risk prostate cancer patients, but a further case–control study is needed.

Many studies have indicated that CCIS is an accurate, flexible, and easily accessible tool for predicting the mortality for patients treated with radical prostatectomy 14, 15, 16, 17. But in our analysis of CCIS and CHADS2 scores, the prediction accuracy for ischemic stroke by CCIS is lower (52.2%) than that obtained using CHADS2 scores (97.8%). We suggest that CHADS2 score provides better predictive accuracy for ischemic stroke in prostate cancer patients.

It would be very important to look to see if the CHADS2 VASc score does better or worse than the CHADS2 score since the CHADS2 VASc score is what is being used clinically now to a much greater extent than the CHADS2 score 12, 13. We also used CHADS2 VASc score to predict ischemic stroke in these patient and the result was showed in Table S1. We did not find better prediction value of CHADS2 VASc score.

Although the findings from this study may potentially affect many patients diagnosed with prostate cancer, several caveats need to be considered. First, the diagnosis of the co‐morbid conditions is completely dependent on ICD‐9‐CM Codes. However, the NHI Bureau randomly reviews patient charts and conducts patient interviews to verify diagnosis accuracy. Hospitals with outlier charges or practices may incur audits, with subsequent heavy penalties for malpractice or discrepancies. Second, the severity of prostate cancer cannot be precisely extracted from ICD‐9‐CM codes, preventing further subgroup analysis. Third, the database does not contain information on daily activity, dietary habits, and body mass index, which may also be risk factors for ischemic stroke. Further studies linking administrative data and primary hospitalization information such as severity of ischemic stroke and detailed risk factors are warranted. On the whole, given the magnitude and statistical significance of the observed effects in this study, these limitations are unlikely to compromise the results.

## Conclusions

In conclusion, the CHADS2 score could serve as an accurate and easily accessible tool to predict the incidence of ischemic stroke in prostate cancer patients treated with radical prostatectomy. Cardiovascular risk evaluation and management should be applied more aggressively for such patients with a CHADS2 score ≥ 2.

## Conflict of Interest

The authors have declared that no competing interests exist.

## Supporting information


**Figure S1.** Receiver operating characteristics curve for CHADS2, CHA2DS2, and CCIS score in prediction of stroke in prostate cancer with radical prostatectomy patients.

**Table S2.** Baseline characteristics of the patients with radical prostatectomy prostate cancer and without prostate cancer from 1997 to 2011 in Taiwan.
