# Supplementary material for: CHADS2 scores as a predictor of ischemic stroke after radical prostatectomy
Source: Cancer Med. 2015 Nov 21;5(1):3–8. doi: 10.1002/cam4.557 (PMC4708895; doi:10.1002/cam4.557)
Supplement: Supplementary file 1 — Figure S1. Receiver operating characteristics curve for CHADS2, CHA2DS2, and CCIS score in prediction of stroke in prostate cancer with radical prostatectomy patients. [file CAM4-5-003-s001.docx]

| 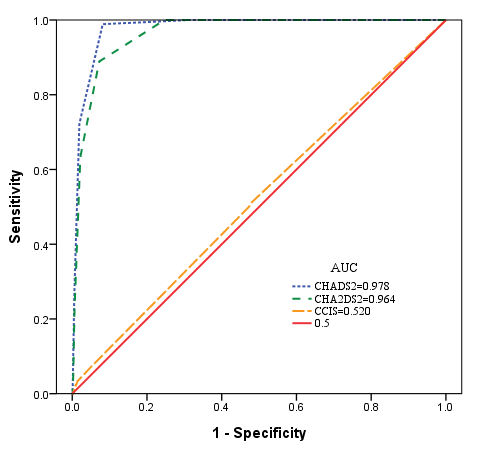 |
| --- |
| **Figure**. Receiver operating characteristics curve for CHADS2,CHA2DS2 and CCIS score in prediction of stroke in prostate cancer with radical prostatectomy patients. |
