# Supplementary material for: CHADS2 scores as a predictor of ischemic stroke after radical prostatectomy
Source: Cancer Med. 2015 Nov 21;5(1):3–8. doi: 10.1002/cam4.557 (PMC4708895; doi:10.1002/cam4.557)
Supplement: Supplementary file 2 — Table S2. Baseline characteristics of the patients with radical prostatectomy prostate cancer and without prostate cancer from 1997 to 2011 in Taiwan. [file CAM4-5-003-s002.docx]

**Supporting Information 1:**

| Baseline characteristics of the patients with radical prostatectomy prostate cancer and without prostate cancer from 1997 to 2011 in Taiwan. | | | |
| --- | --- | --- | --- |
| Variables | prostate cancer | No prostate cancer | p-value |
| Total | 5414 | 10826 |  |
| Mean age,years(±SD) | 65±6 | 65±6 | NA |
| CHADS2 score(mean±SD) | 0.5±0.8 | 0.42±0.94 | 0.006 |
| 0-1 (%) | 4890(90.3) | 9655(89.2) |  |
| 2-3 (%) | 469(8.7) | 912(8.4) |  |
| > 4 (%) | 54(1.0) | 259(2.4) |  |
| CHA2DS2-VASc score(mean±SD) | 1.06±1.05 | 1.02±1.18 | 0.054 |
| 0-1 (%) | 4009(74.1) | 8602(79.5) |  |
| 2-3 (%) | 1232(22.8) | 1594(14.7) |  |
| > 4 (%) | 172(3.2) | 630(5.8) |  |
| Charlson Comorbidity Index Score(mean±SD) | 1.1±1.3 | 0.01±0.13 | <0.001 |
| 0-1 (%) | 2851(52.7) | 10791(99.7) |  |
| 2-3 (%) | 2484(45.9) | 34(0.3) |  |
| > 4 (%) | 78(1.4) | 1(0.01) |  |
| Comborbidities |  |  |  |
| Hyperlipidemia (%) | 139(2.6) | 209(1.9) | <0.001 |
| Chronic kidney disease (%) | 28(0.5) | 68(0.6) | 0.678 |
| Coronary artery disease (%) | 240(4.4) | 705(6.5) | 0.029 |
| Socioeconomic status |  |  | <0.001 |
| Disadvantaged SES (%) | 1395(25.8) | 4227(39.0) |  |
| Advantaged SES (%) | 4019(74.2) | 6599(61.0) |  |
| Urbanization |  |  | <0.001 |
| Urban (%) | 1995(36.9) | 2790(25.8) |  |
| Nonurban (%) | 3418(63.1) | 8036(74.2) |  |
| Geographic region |  |  | <0.001 |
| Northern and Central (%) | 4116(76.0) | 6905(63.8) |  |
| Southern and Eastern (%) | 1297(24.0) | 3921(36.2) |  |
